# Jaboticaba (*Myrciaria jaboticaba*) Attenuates Ventricular Remodeling after Myocardial Infarction in Rats

**DOI:** 10.3390/antiox11020249

**Published:** 2022-01-27

**Authors:** Renata Candido da Silva, Bertha Furlan Polegato, Paula Shmidt Azevedo, Ana Angélica Fernandes, Katashi Okoshi, Sergio Alberto Rupp de Paiva, Marcos Ferreira Minicucci, Leonardo Antônio Mamede Zornoff

**Affiliations:** 1 Internal Medicine Department, Botucatu Medical School, São Paulo State University (UNESP), Botucatu 18618-687, Brazil; renata_candido88@yahoo.com.br (R.C.d.S.); bertha.polegato@unesp.br (B.F.P.); schmidt.azevedo@unesp.br (P.S.A.); katashi.okoshi@unesp.br (K.O.); sergio.paiva@unesp.br (S.A.R.d.P.); minicucci@fmb.unesp.br (M.F.M.); 2 Chemistry and Biochemistry Department, Institute of Biosciences of Botucatu, São Paulo State University (UNESP), Botucatu 18618-687, Brazil; ana.ah.fernandes@unesp.br

**Keywords:** cardiac remodeling, jaboticaba, oxidative stress, fibrosis, energy metabolism

## Abstract

The cardiac remodeling after myocardial infarction is characterized by inflammation and oxidative stress. Thus, this study aimed to test the hypothesis that jaboticaba, due to its anti-inflammatory and antioxidants properties, attenuates cardiac remodeling after myocardial infarction. Wistar rats were submitted to myocardial infarction due to coronary artery occlusion, and divided into four experimental groups: C, sham control animals; I, animals submitted to myocardial infarction, received a standard diet; IJ2, animals submitted to myocardial infarction, received a standard diet plus 2% jaboticaba; and IJ4, animals submitted to myocardial infarction, received a standard diet plus 4% jaboticaba. After a three-month follow-up, echocardiography, histology, oxidative stress, and cardiac energy metabolism were analyzed. There was no difference in infarct size or mortality among the infarcted groups. The IJ4 group displayed improved diastolic function, as assessed by isovolumetric relaxation time normalized to the heart rate. As expected, the percentage of collagen was higher in all infarcted groups than in the C group. However, the IJ2 group had less collagen than groups I and IJ4. The IJ4 group presented lower PFK activity than I and IJ2, and lower pyruvate dehydrogenase activity than controls, whereas the IJ2 group showed no differences compared to the control group in both LDH and ATP synthase activity. The 2% and 4% doses attenuated lipid peroxidation and increased the activity of glutathione peroxidase compared with the I group. In conclusion, jaboticaba attenuated the remodeling process after myocardial infarction, which was associated with decreased oxidative stress and improved energy metabolism.

## 1. Introduction

After myocardial infarction (MI), complex changes in ventricular architecture can occur, including cavity dilation, hypertrophy, and fibrosis of non-infarcted regions. This overall process of alterations in ventricular size, composition, and mass is known as ventricular remodeling [1,2,3]. Importantly, cardiac remodeling after MI is associated with a poor prognosis, mainly owing to the development of heart failure. Although several strategies are already consolidated in the prevention of remodeling, this process continues to occur in a large number of patients after coronary occlusion [4]. Therefore, new strategies to attenuate this process are critical in this clinical setting.

Several mechanisms modulate cardiac remodeling after MI, including neurohormonal activation, increase in cell death by apoptosis and autophagy, alterations in the contractile proteins, alterations in the calcium transport system, collagen accumulation, changes in matricellular proteins, metalloproteases activation, inflammation, oxidative stress, and changes in energy metabolism [1,2,3]. Importantly, after infarction, cardiac remodeling has been attenuated by the administration of multiple antioxidant products, including n-acetylcysteine [5], green tea [6], rosemary [7], tomato/lycopene [8], and probucol [9].

Jaboticaba (*Myrciaria jaboticaba*) is a fruit native to Brazil, belonging to the Myrtaceae family, the main components of which are the anthocyanins cyanidin-3-glycoside and delphinidin-3-glucoside, known for their antioxidant and anti-inflammatory properties [10,11]. However, the effects of jaboticaba on cardiac remodeling after MI remain unknown. Thus, this study aimed to test the hypothesis that jaboticaba attenuates cardiac remodeling after myocardial infarction. Our results showed that jaboticaba attenuated the remodeling process after myocardial infarction, which was associated with decreased oxidative stress and improved energy metabolism.

## 2. Material and Methods

All experimental procedures were performed in accordance with the National Institute of Health’s Guide for the Care and Use of Laboratory Animals and approved by the Animal Ethics Committee of our institution.

### 2.1. Experimental Groups

Male Wistar rats, weighing 200–250 g, were subjected to experimental myocardial infarction, according to a previously described method [12,13]. In brief, the rats were anesthetized with an intramuscular injection of ketamine (70 mg/kg) and xylazine (5 mg/kg), and after a left thoracotomy, the heart was exteriorized by lateral compression of the thorax. The left atrium was retracted to facilitate ligation of the left coronary artery with 5-0 mononylon between the pulmonary outflow tract and the left atrium. The heart was returned to the chest, the lungs expanded by positive pressure, the pneumothorax aspirated, and the incision closed.

We selected only animals with an infarct size greater than 35%, as assessed by histology because animals with a small infarct do not experience cardiac remodeling [14].

After 5 days of the surgical procedure to induce infarction, an initial echocardiographic study was performed to guarantee homogeneity between the groups (data not shown).

After this echocardiogram, the animals were allocated into four experimental groups and observed for three months: C (*n* = 16), sham control animals; I (*n* = 37), animals submitted to myocardial infarction, received standard diet; IJ2 (*n* = 36), animals submitted to myocardial infarction, received standard diet plus 2% jaboticaba; and IJ4 (*n* = 37), animals submitted to myocardial infarction, received standard diet plus 4% jaboticaba. All animals were housed in individual cages in a room maintained at 23 °C on a 12:12 h light:dark cycle. Before the 5 days, all animals consumed a standard diet. Rats consumed food and water ad libitum.

### 2.2. Feed

Nuvilab feed (Nuvital^®^) was powdered for use with jaboticaba and pelletized for use as standard feed. The approximate composition per kg of feed is 220 g of protein, 40 g of fat, 100 g of minerals, and 80 g of fiber. Ripe Sabará jaboticaba fruits (*M. jaboticaba* Vell Berg) were used. All jaboticaba used in the experiment was purchased from a local producer. The whole fruit (peel + pulp + seed) was homogenized in an industrial blender, packed in smaller plastic containers, and frozen at −80 °C. The moisture content was 87.3%, which was considered in the calculation of the quantity of jaboticaba used in the supplementation. The feed was stored in a freezer (−20 °C). Animal feed intake per 24 h was controlled. The animals were weighed weekly throughout the experimental period. We supplemented jaboticaba at two doses: 20 g of homogenate/kg (2%) of diet and 40 g of homogenate/kg (4%) of diet, based on a previous study [11].

A rat consumes an average of 25 g of feed per day; thus, the concentrations of 2% and 4% are equivalent to 0.5 and 1.0 g of jaboticaba added to the feed per day, respectively. We used the dose equivalence equation proposed by Reagan-Shaw et al. [15] to calculate the equivalent dose in humans and determined that 0.5 g and 1.0 g of jaboticaba in mice are equivalent to 15.6 g and 31.2 g in humans, which, for convenience, can be converted into one and two tablespoons, respectively.

### 2.3. Physical-Chemical Characterization of Jaboticaba Samples

The total anthocyanin content was measured according to the methodology of Teixeira et al. [16]. The antioxidant activity was determined by the percentage of radical elimination of 2,2-diphenyl-1-picrylhydrazyl (DPPH) in a methanol solution, as described previously [8]. The reduction in DPPH was monitored by the decrease in absorbance at a characteristic wavelength during the reaction. The content of total phenolic compounds was determined using the Folin–Ciocalteu reagent, according to the procedure of Singleton et al. [17]. The sample absorbance was determined at 725 nm after 30 min. The calculations were performed from the standard curve and the results are expressed in mg equivalents of gallic acid per gram of pulp.

### 2.4. Echocardiographic Analysis

Echocardiography was performed after a three-month follow-up, according to previous methods [18,19]. Rats were lightly anesthetized by intramuscular injection with a mixture of ketamine (50 mg/kg) and xylazine (1 mg/kg). All tracings were manually measred with a caliper by the same observer. LV end-diastolic dimension (LVEDD) and posterior wall thickness were measured at maximal diastolic dimension, and the end-systolic dimension (LVSD) was measured at the point of maximal anterior motion of posterior wall. The systolic (SA) and diastolic areas (DA) were measured in two dimensions using planimetry. The fractional area change (FAC = DA − SA/DA × 100), and ejection fraction assessed LV systolic function, E wave deceleration time, and isovolumetric relaxation time normalized to the heart rate (IRT/RR^0.5^) assessed diastolic function.

### 2.5. Morphometric Analysis

The morphometric analysis of the myocardium was performed as described previously [20]. Briefly, the myocyte cross-sectional area (CSA) was determined using hematoxylin and eosin staining, and the interstitial collagen volume fraction through picrosirius red staining using an automated image analyzer. All measurements were performed using a Leica microscope (lens magnification 400×) attached to a video camera and connected to a personal computer equipped with image analyzer software (Image-Pro Plus 3.0, Media Cybernetics, Silver Spring, MD, USA).

Planimetry determined the lengths of the infarcted and viable muscles for both the endocardial and epicardial circumferences. Infarct size was determined by dividing the endocardial and epicardial circumferences of the infarcted area by the total epicardial and endocardial ventricular circumferences. We performed the measurements on ventricular sections (5–6 mm from the apex) considering that the left mid-ventricular slice has a close linear relationship with the sum of the area of all heart sections [21].

### 2.6. Cardiac Energy Metabolism and Oxidative Stress Analysis

Analysis was performed in cardiac tissue, above 5 mm from the apex. The activities of lactate dehydrogenase (LDH), citrate synthase (CS), β-hydroxyacyl-CoA dehydrogenase (β-OH-acyl-CoA DH), phosphofructokinase (PFK), pyruvate dehydrogenase complex (PIDH), NADH dehydrogenase (complex I), succinate dehydrogenase (complex II), and ATP synthase were measured using a previously described method [21].

Oxidative stress was assessed by determining the concentration of lipid hydroperoxide and carbonyl protein. Glutathione peroxidase (GSH-Px), superoxide dismutase (SOD), and catalase (CAT) activities were assessed as previously described [21].

### 2.7. Western Blot Analysis

Briefly, in this analysis, 60 mg of the non-infarcted left ventricle were used. Nuclear protein extraction was performed with buffer (10 mM HEPES, 1.5 mM MgCl_2_, 10 m MKC_l_, 0.5 mM DTT, 0.05% NP40). The supernatant was discarded (cytoplasmic fraction) and the pellet resuspended with buffer (5 mM HEPES, 1.5 mM MgCl_2_, 0.2 mM EDTA, 0.5 mM DTT, 26% glycerol (*v*/*v*)); NaC_l_ was added, then the result was homogenized. The samples were manually homogenized with the aid of a glass rod. After waiting 30 min on ice, samples were centrifuged again at 15,000 rpm for 20 min at 4 °C and the supernatant (nuclear fraction) was collected and used for the quantification of Nrf-2. The electrophoretic run and next steps were the same as used for Western blot previously described (primary antibody Nrf-2, rabbit polyclonal IgG, Santa Cruz Biotechnology, Inc., Heidelberg, Germany, sc-722; 1:400. Secondary antibody goat anti-rabbit IgG-HRP, Santa Cruz Biotechnology, Inc., Heidelberg, Germany, sc 2004; 1:8000) [22].

### 2.8. Statistical Analysis

The data are expressed as mean ± standard deviation (for normal distribution) or median with the 25th and 75th percentiles (for non-normal distribution). Continuous variables were tested for normality; continuous variables with normal distribution were compared by ANOVA and the Holm-Sidak test, whereas non-normal continuous variables were compared by Kruskal-Wallis and Dunn tests. Data analysis was performed using SigmaStat for Windows v2.03 (SPSS, Inc., Chicago, IL, USA). The significance level was set at 5%.

## 3. Results

We used 205 animals: 16 sham and 189 infarcted animals. After 5 days, 51 rats died and, after 3 months, 9 animals died. Additionally, 46 animals were discarded with an infarct size of <35%. The sham group did not present any deaths. Therefore, our final groups were composed of the following animals: C = 16, I = 14, IJ2 = 23 and IJ4 = 18.

There was no difference in infarct size (I = 43 ± 00B1 7%, IJ2 = 44 ± 5%, and IJ4 = 43 ± 5%; *p* > 0.05) nor mortality among the infarcted groups 3 months after infarction (I = 3, IJ2 = 3 and IJ4 = 3; *p* > 0.05).

We analyzed the physical-chemical characterization of the jaboticaba samples. The content of total phenolic compounds was 532 ± 8 (mg/100 g), antioxidant activity was 248 ± 1 (DPPH/kg), and content of total anthocyanins was 153 ± 6 (mg/100 g).

The animals were weighed weekly and feed was consumed daily. There was no significant difference in the initial and final body weights nor the average daily consumption, in grams, of feed among the groups (Table 1).

The results of the echocardiographic study are shown in Table 2. Myocardial infarction induced an increase in the size of the left cardiac chambers, accompanied by changes in systolic and diastolic function. The IJ4 group exhibited improved diastolic function, as assessed by IRT/RR^0.5^, in comparison to I and IJ2. However, jaboticaba did not affect other variables.

**Figure 1:**
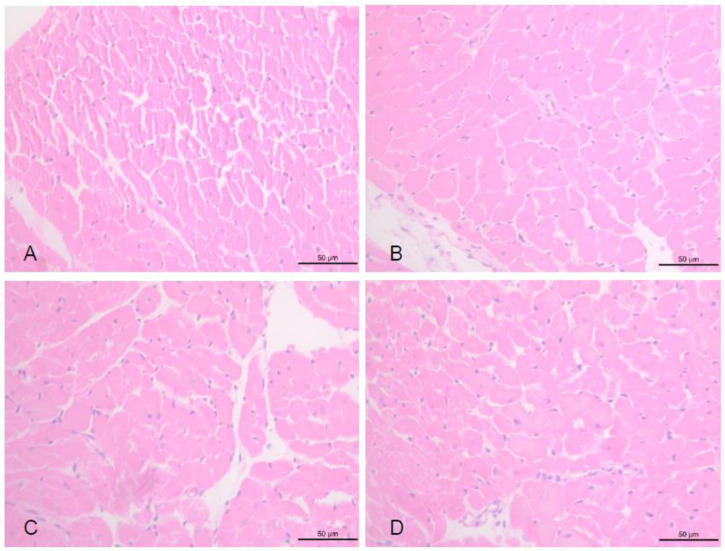
Myocyte Cross-Sectional Area. (**A**) control animals; (**B**) infarcted animals; (**C**) infarcted animals supplemented with jaboticaba 2%; (**D**) infarcted animals supplemented with jaboticaba 4%.

In the histological study, infarction increased the CSA of myocytes (Figure 1). Jaboticaba did not affect this variable (C = 362 ± 79 µm^2^, I = 673 ± 72 µm^2^, IJ2 = 624 ± 91 µm^2^ and IJ4 = 629 ± 77 µm^2^; *p* > 0.05).

**Figure 2:**
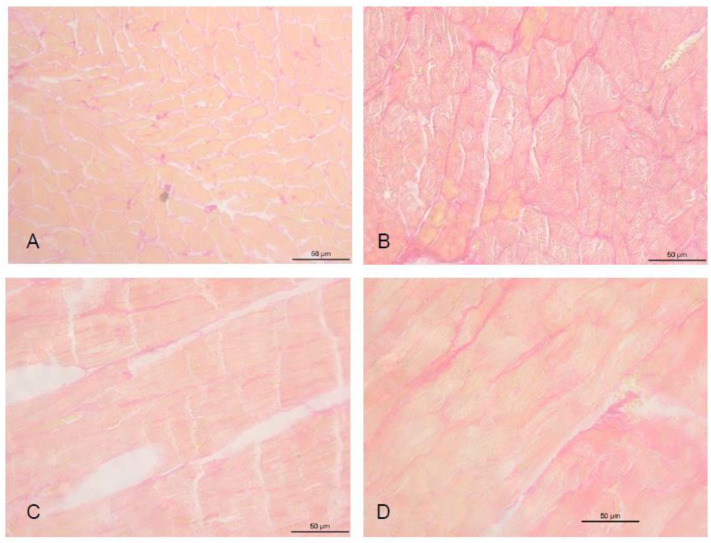
Interstitial Collagen Volume Fraction. (**A**) control animals; (**B**) infarcted animals; (**C**) infarcted animals supplemented with jaboticaba 2%; (**D**) infarcted animals supplemented with jaboticaba 4%.

The percentage of collagen was higher in all infarcted groups than in the C group (Figure 2). However, the IJ2 group had less fibrosis than groups I and IJ4 (C = 2.81 ± 0.87%, I = 7.82 ± 0.55% *, IJ2 = 5.82 ± 0.17%, IJ4 = 7.69 ± 0.15% *; * *p* < 0.05 versus C and IJ2).

The results for cardiac energy metabolism are shown in Table 3. Infarction resulted in higher LDH and PKF and lower CS, β-OH-acyl-CoA DH, ATP synthase, complex I, and complex II activities. The IJ4 group presented lower PFK activity than I and IJ2, and lower pyruvate dehydrogenase activity than controls, whereas the IJ2 group showed no differences compared to the control group in both LDH and ATP synthase activity.

The results of the biochemical assessment of oxidative stress are shown in Table 4. The infarction induced high levels of carbonyl protein and supplementation with jaboticaba did not change this. The infarction resulted in a higher concentration of lipid hydroperoxide and lower catalase, SOD, and GSH-Px activity than observed in the C group. Regarding the influence of jaboticaba supplementation, the 2% and 4% doses attenuated lipid peroxidation and increased the activity of GSH-Px compared with the I group, with no difference between the doses.

**Figure 3:**
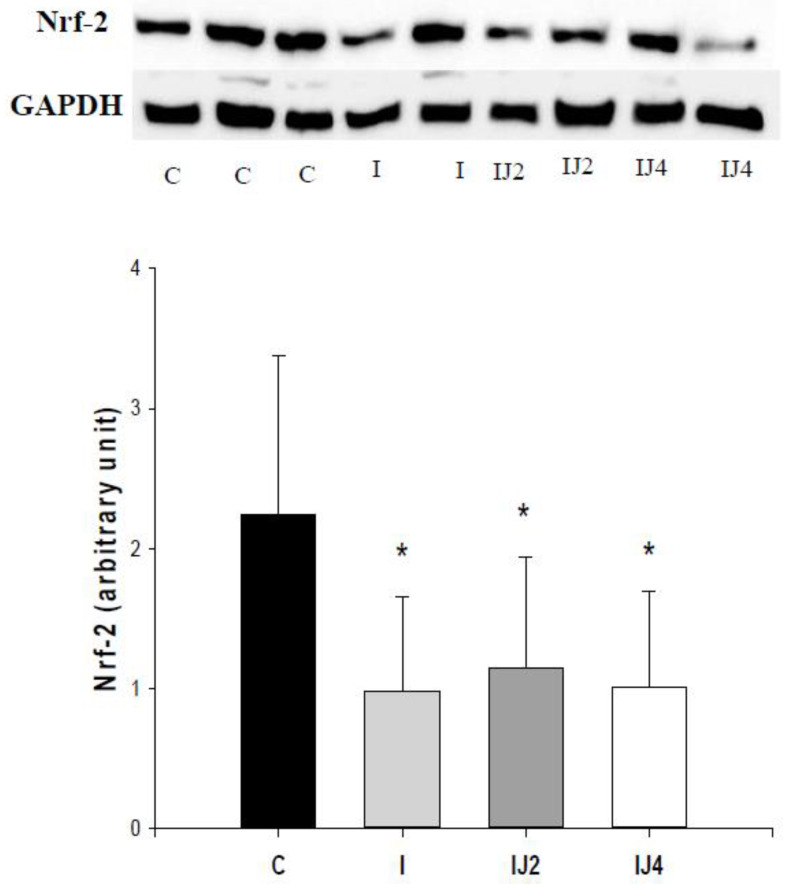
Nrf-2 levels. C: control animals; I: infarcted animals; IJ2: infarcted animals supplemented with jaboticaba 2%; IJ4: infarcted animals supplemented with jaboticaba 4%. * *p* < 0.05 versus C.

Myocardial infarction decreased Nrf-2 levels and jaboticaba did not affect this (C = 2.24 ± 1.14, I = 0.98 ± 0.67 *, IJ2 = 1.14 ± 0.80 *, IJ4 = 1.01 ± 0.68 *; *p <* 0.05 vs. C), as showed in Figure 3.

## 4. Discussion

This study aimed to evaluate the influence of jaboticaba supplementation on cardiac remodeling after myocardial infarction. Our data show that, as expected, infarction induced morphological and functional cardiac changes, which were associated with oxidative stress and a worsening of energy metabolism. Importantly, jaboticaba attenuated oxidative stress, improved energy metabolism, decreased fibrosis in the non-infarcted region, and improved diastolic function. Thus, our results indicate that jaboticaba attenuated the remodeling process after coronary occlusion.

Jaboticaba, often used to make jams and liqueurs, is also used in popular medicine, mainly in southern Brazil, for its antioxidant actions and in the treatment of spasmodic vasomotor disorders [23,24]. Many phenolic compounds, flavonoids, and anthocyanins are present in the fruit, which are responsible for its biological and potentially medicinal effects. One of the main effects of jaboticaba is its antioxidant action but some studies have described its anti-inflammatory, anticancer, and anti-aging actions [25]. Additionally, some studies have evaluated the effects of jaboticaba supplementation on the cardiovascular system and have shown a vasorelaxant effect, probably via the activation of K^+^ channels and inhibition of Ca^2+^ influx [26,27]. However, the actions of jaboticaba in the cardiac remodeling process after myocardial infarction remain unknown.

The first aspect to be considered is that cardiac remodeling is an extremely complex event characterized by genetic, molecular, and cellular changes. Despite this complexity, remodeling is clinically diagnosed by changes in cardiac morphology and function [1,2,3]. In this sense, one of the main changes after infarction is the accumulation of collagen in non-infarcted regions. Importantly, our results showed that jaboticaba decreased cardiac fibrosis, which was associated with improved diastolic function. Therefore, jaboticaba attenuated the clinical manifestations of the remodeling process after infarction.

One of the main modulators of the remodeling process is cardiac energy metabolism. After injury, the preferential use of fatty acids, observed in normal hearts, can be substituted with that of glucose, and other changes in energy homeostasis also occur [28,29]. Additionally, in ischemic conditions, the anaerobic metabolism of carbohydrates occurs in the heart, which leads to the formation of lactate and, consequently, an increase in LDH activity [30]. Furthermore, the activity of citrate synthase, the initial enzyme in the citric acid cycle, may be reduced in heart failure owing to the inability of the mitochondria to transport electrons and perform oxidative phosphorylation, thereby compromising energy production [31]. In our study, supplementation with jaboticaba decreased the activity of different enzymes in the glycolytic pathway, which was associated with improved mitochondrial function, as evidenced by the increase in the activity of ATP synthase. Therefore, we can conclude that jaboticaba attenuated different changes in cardiac energy metabolism induced by infarction.

Oxidative stress and redox signaling are important contributors to cardiac remodeling [32]. Under physiological conditions, the toxic effects of reactive oxygen species can be partially prevented by an enzyme system, which includes GSH-Px, SOD, and CAT [33]. Nrf-2 is a transcription factor that binds to the promoter region of several genes involved in the antioxidant response [34,35]. Under normal conditions, Nrf-2 is retained in the cytoplasm. However, in stressful situations, Nrf-2 migrates to the nucleus and combines with a small protein, forming a heterodimer and initiating the transcription of genes involved in the antioxidant response [36]. In our study, there was no effect of jaboticaba on Nrf-2 expression; however, supplementation with jaboticaba increased the activity of GSH-Px compared with the I group, which suggested that jaboticaba improved the endogenous antioxidant response.

Lipid peroxidation is defined as a chain reaction that destroys the lipid membrane and causes changes in cell structure and permeability. It begins with the sequestration of hydrogen from the lipid membrane, forming hydroperoxides, which are considered biomarkers of damage induced by reactive oxygen species [37] Furthermore, the action of reactive oxygen species causes protein damage, which results in the generation of carbonyl groups, aldehydes, and ketones. Thus, carbonyl protein is a biomarker of oxidative damage to proteins [38]. In our study, infarction induced oxidative stress, as expected. Although supplementation with jaboticaba did not affect protein damage, it decreased lipid peroxidation. Therefore, we can conclude that jaboticaba attenuated the oxidative stress induced by myocardial infarction.

An important issue is that jaboticaba has bioactive compounds, such as polyphenols and anthocyanins, concentrated in its purple bark, in addition to quercetin and proanthocyanidin derivatives. Additionally, they also contain ellagic acid derivatives. The main anthocyanins characterized, especially in jaboticaba bark, are cyanidin-3-glucoside and delphinidin-3-glycoside, known for their antioxidant and anti-inflammatory properties. Ellagic acid and its derivatives also have antioxidant activity and other beneficial biological effects, such as antiproliferative and cardioprotective [39,40]. In a study carried out by Pinto et al., it was found that ellagic acid is capable of inhibiting the angiotensin I-converting enzyme, recognized as a modulator of the cardiac remodeling process [11]. Thus, we believe that these components may have been the modulators of jaboticaba effects in our study.

Finally, we must consider that some effects of jaboticaba were obtained only with the double dose, whereas other effects were observed only with the single dose. This phenomenon suggests that specific targets of the cardiac remodeling process are sensitive to different doses of jaboticaba; however, this needs to be confirmed in future studies.

## 5. Conclusions

In conclusion, jaboticaba attenuated the remodeling process after myocardial infarction, as measured by decreased fibrosis in the non-infarcted region and improved diastolic function, which were associated with decreased oxidative stress and improved energy metabolism.

## Figures and Tables

**Table 1:** Initial and final body weight and daily feed intake.

Variables	C (*n* = 16)	I (*n* = 14)	IJ2 (*n* = 23)	IJ4 (*n* = 18)
Initial body weight (g)	251 ± 19	239 ± 16	247 ± 23	245 ± 24
Final body weight (g)	379 ± 30	388 ± 25	400 ± 35	386 ± 32
Feed intake (g/day)	20 (19–22)	21 (19–22)	20 (19–21)	19 (18–20)

C: control animals; I: infarcted animals; IJ2: infarcted animals supplemented with jaboticaba 2%; IJ4: infarcted animals supplemented with jaboticaba 4%. Data are expressed as the mean ± SD or median (including the lower quartile and upper quartile). *p* > 0.05.

**Table 2:** Echocardiographic data.

Variables	C	I	IJ2	IJ4
LA (mm)	5.4 ± 0.6	6.2 ± 0.9 *	6.5 ± 0.8 *	6.4 ± 0.8 *
LVDD (mm)	7.1 ± 0.7	9.2 ± 0.5 *	9.3 ± 0.7 *	9.4 ± 0.7 *
LVSD (mm)	2.8 ± 0.6	7.0 ± 0.6 *	7.1 ± 0.8 *	7.1 ± 0.9 *
EF (%)	0.9 ± 0.1	0.5 ± 0.1 *	0.5 ± 0.1 *	0.6 ± 0.1 *
FAC (%)	77 ± 6.7	35 ± 7.7 *	33 ± 7.6 *	32 ± 12 *
EDT	43 ± 7.1	47 ± 6.8 *	51 ± 11 *	51 ± 10 *
IRT/RR^0.5^	52 ± 7.0	62 ± 10 *	64 ± 9.5 *	59 ± 10

C: control animals; I: infarcted animals; IJ2: infarcted animals supplemented with jaboticaba 2%; IJ4: infarcted animals supplemented with jaboticaba 4%. LA, left atrium diameter; LVDD, left ventricular (LV) diastolic diameter; LVSD, LV systolic diameter; EF, ejection fraction; FAC, fractional area change; EDT, E wave deceleration time; IRT/RR, isovolumetric relaxation time normalized by heart rate. Data are expressed as the mean ± SD. All animals were subjected to an echocardiogram. * *p* < 0.05 versus C.

**Table 3:** Cardiac energy metabolism assessment.

Variables	C (*n* = 8)	I (*n* = 8)	IJ2 (*n* = 8)	IJ4 (*n* = 8)
Phosphofructokinase (nmol/g)	148 ± 29	355 ± 78 *^#^	340 ± 99 *^#^	134 ± 62
Pyruvate dehydrogenase complex (nmol/g)	282 ± 30	212 ± 56	244 ± 35	186 ± 37 *
Lactate dehydrogenase (nmol/g)	61 ± 10	92 ± 28 *	78 ± 14	91 ± 24 *
β-Hydroxyacyl-CoA dehydrogenase (nmol/mg)	26 ± 5	10 ± 3 *	8 ± 2 *	9 ± 3 *
Citrate synthase (nmol/g)	31 ± 6	16 ± 3 *	17 ± 3*	16 ± 3 *
Complex I (NADH dehydrogenase) (nmol/mg)	10 ± 1	6 ± 1 *	5 ± 1 *	5 ± 1 *
Complex II (Succinate dehydrogenase) (nmol/mg)	4.87 ± 0.88	2.75 ± 0.54 *	2.88 ± 0.98 *	2.55 ± 0.48 *
ATP synthase (nmol/mg)	30 ± 5	18 ± 4 *	23 ± 4	16 ± 4 *

C: control animals; I: infarcted animals; IJ2: infarcted animals supplemented with jaboticaba 2%; IJ4: infarcted animals supplemented with jaboticaba 4%. Data are expressed as the mean ± SD. * *p* < 0.05 versus C; ^#^ *p* < 0.05 versus IJ4.

**Table 4:** Oxidative stress assessment.

Variables	C (*n* = 8)	I (*n* = 8)	IJ2 (*n* = 8)	IJ4 (*n* = 8)
LH (nmol/mg of tissue)	244 ± 34	325 ± 45 *	289 ± 42	234 ± 39
CP (nmol/mg of protein)	2.8 ± 0.4	4.7 ± 0.6 *	4.4 ± 0.9 *	4.9 ± 0.8 *
CAT (µmol/g of tissue)	53 ± 6.8	37 ± 5.2 *	34 ± 6.4 *	34 ± 7.4 *
SOD (nmol/mg of tissue)	15 ± 3.1	11 ± 1.3 *	9.9 ± 1.8 *	11 ± 1.9 *
GSH-Px (nmol/mg of tissue)	41 ± 8.9	28 ± 6.7 *	43 ± 9.2	41 ± 8.3

C: control animals; I: infarcted animals; IJ2: infarcted animals supplemented with jaboticaba 2%; IJ4: infarcted animals supplemented with jaboticaba 4%. LH: lipid hydroperoxide; CP: carbonyl protein; CAT: catalase; SOD: superoxide dismutase; GSH-Px: glutathione peroxidase. Data are expressed as the mean ± SD. * *p* < 0.05 versus C.

## Data Availability

The authors confirm that the data supporting the findings of this study are available within the article.
